# Comparison of Physicochemical Properties of Bee Pollen with Other Bee Products

**DOI:** 10.3390/biom9120819

**Published:** 2019-12-03

**Authors:** Vaida Adaškevičiūtė, Vilma Kaškonienė, Paulius Kaškonas, Karolina Barčauskaitė, Audrius Maruška

**Affiliations:** 1 Faculty of Natural Sciences, Instrumental Analysis Open Access Centre, Vytautas Magnus University, LT-44404 Kaunas, Lithuania; vaida.adaskeviciute@vdu.lt (V.A.); audrius.maruska@vdu.lt (A.M.); 2 Institute of Metrology, Kaunas University of Technology, LT-51368 Kaunas, Lithuania; paulius.kaskonas@ktu.lt; 3 Lithuanian Research Centre for Agriculture and Forestry, Instituto av.1, LT-58344 Akademija, Kėdainiai District, Lithuania; karolina.barcauskaite@lammc.lt

**Keywords:** bee products, minerals, oxidation-reduction potential, conductivity, hierarchical cluster analysis, antioxidant activity

## Abstract

The aim of this study was to compare the physichochemical composition of various bee products, namely, bee pollen, beebread, propolis, honey, and royal jelly. The samples (37 out of 53) were collected in Lithuania, several samples from other Europe countries (Italy, Denmark, Sweden, Slovakia, Poland, Spain, Republic of Malta, The Netherlands, Latvia, Ukraine) were used for comparison. Various quantities, such as pH, electrical conductivity, oxidation-reduction potential, NaCl content, refraction index, Brix value, total phenolic compound content, total flavonoid content and antiradical activity were measured. Together with the mentioned, the content of micro- and macroelements (As, Ba, Ca, Cd, Co, Cr, Cu, Fe, K, Mg, Mn, Na, Ni, P, Pb, Se, Sr, V and Zn), ultraviolet-visible spectroscopy absorption spectra were analysed. To our knowledge, the literature data about comprehensive comparison of various characteristics of bee products are scarce. Also, to the best of our knowledge, this is the first study revealing mineral content in Lithuanian bee pollen, beebread and royal jelly. The study exposed that bee pollen not only showed the highest values of pH, electrical conductivity and content of soluble solids, but also distinguished from the other samples by the highest flavonoid content (up to 48.3 mg/10 g), the absence of Cr, the presence of Co (0.011–0.100 mg/kg) and Sr (0.73–5.37 mg/kg) and the highest content of Ca (997–2455 mg/kg) and Mg (644–1004 mg/kg). Hierarchical clustering analysis was applied to group the tested samples according to the physicochemical analysis results and mineral content. The clustering analysis revealed that bee pollen formed separate group with the highest distance from the other samples in both cases.

## 1. Introduction

During the last few years, interest in ecological, more functional, balanced, and healthier food products has been increasing rapidly. More and more food industry companies attempt to find some natural food source with high nutritional value with potential of health promotion, reduction of illnesses, etc. Therefore, some food manufacturers pay more attention to apiculture products, namely, pollen, honey, beebread, propolis or royal jelly. Nowadays, these products are considered a functional food, which increase nutritional value and have positive manner to physiological or psychological health [1].

According to the data of the researches, bee products are highly rich in bioactive and nutrition compounds as proteins, minerals, nucleic and amino acids, polyphenols, carbohydrates, phytosterols, vitamins, sugars or minerals. Medicinal importance of bee products has been known since the ancient times. Currently, the chemical profile of these products represents biological properties—anti-inflammatory, antiatherosclerotic, antimicrobic, antioxidant or anticarcinogenic activities [2,3].

Bee pollen is considered an increasingly popular food supplement. This natural product is the result of flower pollen agglutination using nectar or honey and bees’ secreted glands [4]. The significant number of bioactive compounds, carbohydrates, enzymes, vitamins, fatty acids, essential amino acids or carotenoids depends on bee pollen botanical and geographical origin. The variability of metabolites makes this product distinguishable from other bee products and usable in a wide range of medical and therapeutic applications [5]. Also, the composition of nutritional compounds (high amounts of lipids, proteins, carbohydrates) or minerals (Ca, Mg, Fe, Zn, Cu) indicates that pollen could be considered as valuable food and feed [6,7]. The studies of the past few years suggest that bee pollen biologically active substances—apigenin, quercetin, phenil acetic acid, caffeic acid, cinnamic acid, etc., can act as strong antimicrobial, antioxidant, anticarcinogenic, cardioprotective, hepatoprotective or detoxifying sources. Moreover, it is determined that daily use of this product can help to improve eye, skin, cardiovascular or colon functions [7].

Relatively similar composition and properties to bee pollen exhibit one of the sweetest and most flavourful bee products—honey, which is produced by honeybees from the nectar of flowering plants or secretion of living parts of plants. The variability of chemical composition and physical parameters is influenced by its floral source, environmental factors (e.g., temperature), amount of sun or water and geographical origin. Strong medical effect of this bee product originates from the richness of bioactive compounds. High amounts of phenolic acids and flavonoids—pinobanksin, quercetin, kaempferol, coumaric acid stimulate antioxidant, antimicrobial, anticarcinogenic, antiviral and anti-inflammatory activities [3,8]. Also, consumption of this natural product helps to facilitate symptoms of gastroesophageal reflux disease although honey exhibits low pH values [9]. 

Beebread is a product of lactic acid fermentation of bee collected pollen. This unique product distinguishes by higher nutritional value and better digestibility than bee pollen, because during the fermentation process, the walls of pollen cells are partly destructed [10]. According to scientific studies, the chemical composition of beebread is biochemically similar to pollen from which it was made. However, beebread contains more carbohydrates and enzymes, it is richer in content of vitamins K and B, but has less proteins and fats and shows lower pH values because of the lactic acid [11,12]. This natural product owing to its biochemical diverse could be used for immunity system enhancement, regulation of digestive system function, antimicrobial, anti-aging and anti-anemic activities. Furthermore, it has a positive influence on functions of endocrine and nervous systems, tissue regeneration and elimination of various toxins forms [12,13].

Propolis is a mixture of saliva and beeswax with bee collected parts, fluids and flower buds of plants. Due to its specific physical and chemical properties, bees use this material as glue to construct or repair the hive and protect against attempters [14]. Since ancient times, this natural product has been used as a drug against flue, upper respiratory tract infections, dermatological problems (burns, acne, herpes or neurodermatitis), gingivitis or stomatitis. Nowadays, propolis is widely popular in cosmetics, mouthwashes or toothpastes due to its antioxidant and antibacterial properties [15]. The pharmacological properties of propolis are determined by a significant number of steroids, carbohydrates, flavonoids (quercetin, kaempferol, naringenin, galangin, etc.), phenolic acids (caffeic, gallic, vanillic acids, etc.), terpenoids, amino acids, ketones, and vitamins. Therefore, scientific studies of physical and chemical composition of propolis suggest to use this natural product as an official medicine [16,17].

One of the most important food for honeybee larvae and queen during all its larval phase is royal jelly, known as bee’s milk. This white viscous liquid is produced by hypopharyngeal and mandibular glands of old bee workers [18]. Royal jelly has a more consistent composition than honey, pollen or other bee products. Typically. chemical composition is characterised by large amount of water, proteins, sugars, lipids and vitamins. This creamy product is also rich in amino acids (valine, glycine, proline, methionine and tyrosine) and minerals (potassium, calcium, phosphorus, manganese, iron) [19]. Very important role in royal jelly chemical composition plays phenolic compounds and flavonoids, which determine antioxidant and antibacterial effect. Moreover, scientists have determined that usage of this viscous product shows a positive result against tumours (especially leukaemia) and chronic diabetes [9,20].

As it could be seen bee products are gifted with variety of nutritional and bioactive compounds. The aim of this research was to analyse and compare bee pollen and other bee products (propolis, honey, beebread and royal jelly) physicochemical properties using instrumental analysis methods. Obtained data will help to properly characterise these products and determine the main differences between their properties.

## 2. Materials and Methods

### 2.1. Samples

Physicochemical and comparative analysis of 18 samples of dried bee pollen, 11 samples of honey, 10 samples of propolis, eight samples of dried beebread and six samples of royal jelly was performed in this study. The information about tested samples and samples codes used in this paper are listed in Table 1. The most of the samples were collected during flowering season from May to September in Lithuania in 2018. Several commercially available samples of bee pollen, honey and propolis were origin from other European countries. Bee pollen, propolis, beebread and honey samples were stored in a refrigerator at 6 °C for a maximum of four weeks, while royal jelly was kept at –18 °C until analysis. Samples were homogenised with a pestle and porcelain mortar before analysis and extract preparation procedure.

### 2.2. Chemicals and Reagents

Hexametiltetraamine (≥99%) and aluminum chloride (98%) were obtained from Carl Roth Gmbh & Co Kg (Karlsruhe, Germany). The 2,2-diphenyl-1-picrylhydrazyl (DPPH) (99%), methanol (≥99.9%), rutin (95%), hydrochloric (≥37%) and nitric (≥65%) acids of analytical grade were obtained from Sigma-Aldrich Corporation (Taufkirchen, Germany). Folin-Ciocalteu reagent was supplied by the Merck (Darmstadt, Germany). Acetonitrile (99.8%) was obtained from Avantor Performance Materials (Gliwice, Poland). Sodium carbonate and acetic acid (99.9%) were bought from Reachem S. r. o. (Bratislava, Slovakia). The standard mixture solution of multiple microelements (As, Ba, Ca, Cd, Co, Cr, Cu, Fe, K, Mg, Mn, Na, Ni, P, Pb, Se, Sr, V and Zn) in 2% nitric acid was obtained from CPAchem (Bulgaria). Bidistilled water was prepared by means of distillation apparatus Thermo Scientific (Fremont, CA, USA).

### 2.3. The pH Measurement

The pH of samples was measured with pH-meter UltraBasic Benchtop UB-10 (Denver Instrument Company, Denver, CO, USA) with glass electrode. Honey (2 g) or royal jelly (2 g) were dissolved in 15 mL of bidistilled water before analysis. Bee pollen, beebread and propolis samples (each 2 g) were extracted with 15 mL of bidistilled water for 24 h at room temperature. Prepared solutions were filtered through a 0.45 µm polyvinylidene fluoride (PVDF) membrane filter (BGB Analytik, Alexandria, VA, USA). Calibration of pH-meter was performed with three different buffer solutions having pH values of 4, 7 and 10 [21].

### 2.4. Electrical Conductivity

Electrical conductivity of bee products was measured in solutions containing 20% (*w/v*) of dry matter in bidistilled water after filtration with a 0.45 µm membrane filter. Conductivity was measured at 22 °C using WTW inoLab Cond 730 conductometer. Results were expressed as micro Siemens per centimetre (µS/cm) [21].

### 2.5. Refractometry

Bee product under test (2 g) was mixed with 8 mL of bidistilled water and macerated for 24 h. The prepared solutions were centrifuged at 10,000× g for 15 min and filtered through a 0.45 µm PVDF membrane filter. Content of soluble solids (Brix), refraction index (RI) and amount of NaCl was measured by applying four drops of clear extract onto a digital refractometer (Kern & Sohn Gmb ORF-3SM, Balingen, Germany). Content of soluble solids and amount of NaCl were expressed as a percentage [22].

### 2.6. Evaluation of Oxidation-Reduction Potential

An amount of 1.60 g of tested samples was placed in a vial with 6.4 mL of bidistilled water. Honey and royal jelly were filtered with a 0.45 µm membrane filter and oxidation-reduction potential (ORP) was measured immediately, while other products left for extraction for 24 h of separation and then filtered [23]. The ORP was measured with a multimeter having a combined redox electrode (XS Instruments DHS Bench, Reicholzheim, Germany). The ORP values were calculated according to Alwazeer and Dham [24]:

ORP = Eh − 59·(7 − pH)(1)

where Eh is the measured electrode potential, pH is the measured pH value of the extract. The results were expressed in mV.

### 2.7. Ultraviolet-Visible Scanning Spectrophotometry

Tested bee product (0.50 g) was mixed with 25 mL of bidistilled water and macerated for 24 h. The extracts were filtered through a paper filter (Labbox, Barcelona, Spain) and then through a 0.45 µm PVDF membrane filter. The UV-Vis spectra of bee products were performed using different dilution levels: bee pollen samples were diluted 11 times, beebread 18 times, royal jelly 10 times, propolis 19 times and dilution of three times was used for honey solutions. Absorbance values were recorded with a UV-visible spectrophotometer Shimadzu UV-Vis 1280 (Kyoto, Japan) using 1.0 nm scan pitch, 200–1100 nm scan range in 60 s. For all absorbance measurements Quartz cells (1 cm) were used [25].

### 2.8. Spectrophotometric Evaluation

Total phenolic compound content, total flavonoid content and radical scavenging activity were determined spectrophotometrically in bee products using methodology described in Kaškonienė et al. [26]. For these tests an amount of 1 g of bee product was suspended in 10 mL of bidistilled water. The insoluble products, namely, bee pollen, beebread and propolis, were subjected to traditional maceration extraction for 24 h. Obtained extracts and solutions were filtered with a 0.45 µm membrane filter and used for all spectrophotometric tests described below.

Total phenolic content was estimated by the Folin-Ciocalteu method. Extracts (8 µL) were mixed with 240 µL of 3.5% Na_2_CO_3_ and 8 µL Folin-Ciocalteu reagent (The Merck Group, Darmstadt, Germany). The reaction mixtures were prepared using Inteliwasher 3D-IW8 microplate washer (Biosan Laboratories, Riga, Latvia) and measured at 700 nm wavelength with Hipo MPP-96 spectrophotometer (Biosan Laboratories, Latvia) after 30 min of keeping at 22 ± 2°C temperature. A calibration curve of rutin was prepared (0.1–1 mg/mL). Results are expressed as mg rutin equivalent (RUE) per 10 g of raw sample.

Total flavonoid content was determined using colorimetric stock solution, which consisted of 60 mL of methanol, 3 mL of 33% acetic acid, 12 mL of 5% hexametylentetramine, 9 mL of 10% aluminum chloride and 60 mL of bidistilled water. Each prepared extract (10 µL) was mixed with 240 µL of the stock solution and left for 30 min at 4 °C temperature. After incubation, samples were measured at 407 nm wavelength. Total flavonoid content was evaluated using standard curve of rutin (0.1–1 mg/mL). Results are expressed as mg rutin equivalent (RUE) per 10 g of raw sample.

Radical scavenging activity was determined according to colorimetric reaction using 1,1-diphenyl-2-picrylhydrazyl (DPPH) free radical. Bee product extract (5.9 µL) was mixed with 230 µL of the radical stock solution, which comprised 0.1 M pH 5.5 acetate buffer, acetonitrile and methanol (1:1.25:1.25) and had 0.500 absorbance at 515 nm wavelength. All prepared samples were kept for 15 min at 22 ± 2°C temperature in the dark room and then measured at 515 nm wavelength. Radical scavenging activity was evaluated using a standard curve of rutin (0.1–0.5 mg/mL). Results are expressed as mg rutin equivalent (RUE) per 10 g of raw sample.

### 2.9. Microelements Analysis in Bee Products

Microwave-assisted extraction (MAE) was carried out using a CEM MARS 6^®^ (CEM Coorporation, Matthews, NC, USA) digestion system equipped with 100 mL Teflon vessel. Homogenised sample (0.3 g) was accurately weighed into the Teflon vessel and digested using 10 mL concentrated nitric acid and 2 mL concentrated hydrochloric acid. Prior to digestion, the samples were soaked in acid solution for 30 min at room temperature. Digestion was performed under following conditions: temperature 180 °C, pressure 800 psi, ramp time 20 min, hold time 20 min, microwave power 800 W. Then, the digested sample was cooled down, thoroughly transferred into a 100 mL volumetric flask and diluted using bidistilled water to the mark. Each sample was prepared in triplicate and a blank sample was included in each digestion run. 

Qualitative and quantitative evaluation of microelements was performed using inductively coupled plasma mass spectrometer (ICP-MS). Inductively coupled plasma mass spectrometry was performed under helium collision-cell (He-cell) with kinetic energy discrimination mode to remove polyatomic interferences. Samples were introduced using an autosampler with ASXpress™ rapid uptake module (Cetac ASX-520, Teledyne Technologies Inc., Omaha, NE, USA) through a PEEK nebuliser (Burgener Mira Mist, Mississauga, Burgener Research Inc., Mississauga, Canada). Amounts of analysed elements (As, Ba, Cd, Co, Cr, Cu, Fe, Mn, Ni, P, Pb, Se, Sr, V and Zn) were estimated using external multi-element calibration curve in the range 10–200 μg/L. Calibration curve for Ca, K, Na and Mg was developed in the range 50–1000 μg/L.

### 2.10. Statistical Analysis

Chemometric analysis was performed using Matlab software (The MathWorks. Inc., Natick, MA, USA, version R2016b (9.1.0), 64-bit). The data set representing physicochemical properties was composed of 53 samples (18 bee pollen, 10 propolis, 11 honey, eight beebread and six royal jelly samples), when each of them was described by nine variables (pH, electrical conductance, oxidation-reduction potential, amount of NaCl, refraction index, Brix value, total phenolic compound content, total flavonoid content and antiradical activity) measured five times. For mineral analysis, the second data set was composed of 46 samples (15 bee pollen, 11 honey, eight beebread, six propolis and six royal jelly samples) with 15 variables (Mg, K, Ca, P, Cr, Mn, Fe, Co, Cu, Zn, Sr, Cd, Ba, Pb and Na) measured three times.

Data standardization procedure by centering each variable around zero (i.e., subtracting the mean of the variable) and dividing by its standard deviation was done on both sets before data mining. The successive data processing involved analysis of variances (ANOVA), hierarchical clustering analysis (HCA) and correlation analysis.

Analysis of variances was applied for hypotheses testing to evaluate the significance of differences in the means of observed quantities of the tested bee products at chosen *p* ≤ 0.05.

Hierarchical clustering analysis allowed to present similarity among underlying groups of data using multilevel hierarchical structure, dendrogram, was applied on both prepared data sets—the data set representing physicochemical properties of the samples and the data set built from mineral analysis results. The analysis was carried out with chosen Spearman distance as a pairwise distance measure between pairs of observations and Ward’s linkage rule. These metrics were used trying to maximise a cophetetic correlation coefficient. The cophenetic correlation for a dendrogram tree is defined as the linear correlation coefficient between the cophenetic distances obtained from the tree, and the original distances (dissimilarities) used to construct this tree. Therefore, the cophenetic correlation reveals the adequacy of the built dendrogram representing the dissimilarities among observations. The cut-off level of the tree to set data into clusters was chosen as a percentage of the maximum observed distance.

To evaluate relationship between measured quantities describing samples Pearson’s linear correlation coefficient was assessed at chosen *p* ≤ 0.05.

## 3. Results and Discussion

### 3.1. Physicochemical Properties of Bee Pollen and Other Bee Products

Bee pollen and four others investigated in the research bee products (honey, beebread, propolis and royal jelly) were analyzed according to their physicochemical properties (Table 2). Analysis of variances revealed that the values of pH of the tested bee products were identified as statistically different at *p* ≤ 0.05, except the differences of pH between propolis and honey and between propolis and royal jelly samples, which were not statistically significant at the chosen p level. Bee pollen samples distinguished by the highest pH values (4.30–5.22), while the lowest pH values ranging from 3.57 to 4.06 were determined in royal jelly samples. Other studies in literature showed that the pH values of bee pollen varied from 4.3 to 5.9 [27,28], honey from 3.6 to 5.6 [21,29], propolis from 4.7 to 5.3 [21], beebread from 3.8 to 4.3 [12,13] and royal jelly from 3.4 to 4.5 [30,31]. As it can be seen, the results of this research coincide with the data published by other authors.

The electrical conductivity of all studied bee product samples ranged from 142.8 to 836.4 µS/cm. Performed ANOVA revealed that these differences were statistically significant at *p* ≤ 0.05 not only comparing different bee products, but also different samples of the same product. However, there was no significant difference observed between beebread and royal jelly samples. The highest electrical conductivity values were determined in bee pollen samples and scattered from 444.2 to 836.4 µS/cm with an average of 639 ± 127 µS/cm. The lowest electrical conductivity values were recorded in honey samples (142.8–198.8 µS/cm). Other researchers reported that the electrical conductivity of Brazilian honey ranged from 300 to 1400 µS/cm [28]. The observed differences allowed to assume that electrical conductivity, together with other physicochemical properties of bee products, depend on biological and geographical origin.

The electrical conductivity showed moderate correlation (r = 0.689, *p* < 0.001) with quantity of NaCl. To the best of our knowledge, it is the first study about NaCl quantity analysis in bee products. Higher values of NaCl were determined in bee pollen samples (5.66–11.36%), followed by propolis (5.84–6.94%).

Content of soluble solids (Brix) of bee products in this research was found to be in the range from 8.96 to 31.84%. Analysis of variances analysis proved the significance of differences among the tested bee products at *p* ≤ 0.05. The highest value was acquired in bee pollen samples (22.56–31.83%), while propolis samples showed the lowest amount of soluble solids content (8.96–13.84%). This property depends on dissolved substances (sugars, metals, lipids, amino acids, etc.) and the correlation between it and the amount of NaCl was expected. As results showed, these two quantities had moderate correlation (r = 0.576, *p* < 0.001). No literature data with the evaluation of the content of soluble solids in beebread, royal jelly and propolis were found. Japanese scientists have determined that the content of soluble solids in bee pollen reaches about 10.7% [32]. In our study higher values were obtained, they varied from 16.88 to 18.24%. It indicates high dependence of physicochemical properties of bee products on biological and geographical origin.

Prepared bee product extracts were involved in refraction index measurements. To our knowledge, this is the first study of beebread, propolis and bee pollen refractive index analysis. However, the ANOVA revealed that there was no significant difference (*p* > 0.05) of the refractive index between bee pollen and other bee products samples observed. The majority of bee product samples had refractive index from 1.336 to 1.353, which are comparable to water (1.333). The values in literature are higher comparing to our study and the difference may be dependent on botanical and geographical origin of the samples. Ayvaz [33] found the refractive index in the range from 1.49 to 1.51 in Turkish honey. Italian scientists showed that royal jelly samples had refraction index scattered in the range 1.38–1.40 [34]. 

Oxidation-reduction potential was suggested as a simple and fast method for evaluation of antioxidant capacity [35]. The ORP represents the total content of oxidizing/reducing agents. The lower the concentration of oxidant compounds, the lower the ORP value of the sample, and vice versa. The ORP value is related to the chemical composition of the food or other matrix: the presence of amino acids with thiol group, peptides, amount of reducing sugars, vitamins, number of redox couples (e.g., Fe^3+^/Fe^2+^), the pH value, and the dissolved oxygen content [24]. To our knowledge, this is the first study of the oxidation-reduction potential evaluation of the bee products extracts/solutions. The significantly different means of ORP values in the bee pollen and other products were observed according to ANOVA (*p* ≤ 0.05). However, the observed differences could not be stated as significant at this level of p comparing royal jelly and honey, propolis and bee bread and propolis and bee pollen. The ORP values measured in 20% (*w/v*) solutions indicated a distinctive difference between samples—it varied in the interval between 69.10 and 195.92 mV. The highest values of the ORP were determined in propolis samples (82.20–189.96 mV), while the lowest were observed in the honey (69.26–107.91 mV). The ORP values could be used as good predictors of antioxidant activity. Moderate correlation was determined between ORP and total phenolic compound content (r = 0.489, *p* < 0.001), total flavonoid content (r = 0.312, *p* < 0.001) and radical scavenging activity (r = 0.565, *p* < 0.001). In comparison, ORP in fresh sea buckthorn juice was 252 mV [36], in 100 mM ascorbic acid it was 0.828 mV, while in 100 mM α-tocopherol it was 0.134 mV [35].

### 3.2. Spectrophotometric Analysis of Bee Pollen and Other Bee Products

The content of the most important bioactive substances (phenolic compounds and flavonoids) and radical scavenging activity in bee pollen and other bee product samples are presented in Table 3. Significantly different amounts of total phenolic compound content were identified varying from 2.95 to 99.85 mg RUE/10 g. The highest values of phenolic compounds were determined in propolis (68.03–99.85 mg RUE/10 g), while the lowest was in honey (2.95–10.18 mg RUE/10 g) samples. It is difficult to compare the results of this study with the published data because of the different extract solution and reference compound used for the result expression. According to the literature, the highest total phenolic compound content coinciding with this study were recorded in propolis samples and the lowest found in honey. Other researchers showed that the total phenolic compounds content in ethanolic beebread extracts ranged from 2.5 to 13.7 mg gallic acid equivalents (GAE)/g [37], in 50% ethanolic honey solutions from 0.38 to 0.86 mg GAE/g [38], in ethanolic propolis extracts from 269.6 to 426.9 mg GAE/g [39], in ethanolic bee pollen extract from 7.6 to 25.9 mg GAE/g [40]. In our study lower values were obtained, as water was used as extraction solvent.

Analysis of variances hypothesis testing revealed significant differences of means (*p* ≤ 0.05) of total flavonoid content in bee pollen and other bee products (see Table 3). Total flavonoid content in this research varied from 0.28 to 48.31 mg RUE/10 g. It worth mentioning that bee pollen samples showed the highest total flavonoid content value (10.68–48.31 mg RUE/10g). The lowest total flavonoid content was determined in honey (0.28–8.36 mg RUE/10 g). Bee pollen has been proven as the best source of flavonoid compounds by previous studies [41,42], thus obtained results coincide with these studies.

Radical scavenging activity of bee products in this research was observed from 2.73 to 39.64 mg RUE/10 g. The highest and quite similar activity showed propolis (18.79–39.64 mg RUE/10 g) and bee pollen (16.27–39.40 mg RUE/10 g) samples. The correlation coefficient estimated between the radical scavenging activity and the total phenolic and flavonoid contents was 0.690 (*p* ≤ 0.001) and 0.585 (*p* ≤ 0.05), respectively. Literature data showed that methanolic extracts of bee products exhibited higher radical scavenging activity: 1.07–1.44 mg Trolox (TE)/g in bee pollen, 1.14 mg TE/g in beebread, 0.82–1.24 mg TE/g in royal jelly (all these bee products collected in Lithuania) [43], 39–54 mg TE/g in propolis from Mexico [44] and 0.14–0.52 mg TE/g in honey from Thailand [45].

It is difficult to compare the results of this study with the data of other authors because of the different extract solution and reference compounds used for the result expression. However, the results coincide with the literature data: propolis showed the highest activity of radical scavenging, while honey had the lowest value of this property.

### 3.3. Ultraviolet-Visible Scanning Spectrometry

**Figure 1:**
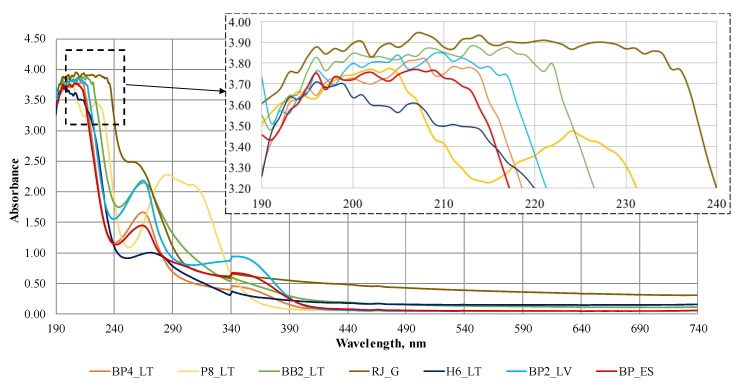
Ultraviolet-Visible spectra of bee pollen and other bee products (sample codes see in Table 1).

The absorbance band presence at a specific wavelength can be considered as presence of a chromophore, which could be identified by using UV-Vis scanning spectrometry. The UV-Vis spectra profile of tested bee products reveals the difference in the chemical composition of the samples (Figure 1). This type of spectrophotometry is dedicated to identify the number and characteristics (position, intensity, shape) of absorption peaks, which help to determine the specific bioactive classes of compounds [46]. The absorbance spectra of all tested bee products were measured in UV-Vis wavelength range from 190 to 740 nm. The spectra profiles of representative samples, which were chosen according to the highest amount of the total phenolic compounds, are shown in Figure 1.

The UV-Vis absorbance spectra of three bee pollen (BP4_LT, BP2_LV, BP_ES), honey (H6_LT), propolis (P8_LT), royal jelly (RJ_G) and beebread (BB2_LT) samples reveal three main regions of peak wavelengths. The peaks in the UV region from 250 to 400 nm uncover the presence of phenolic acids and their derivatives, e.g., flavones, flavonols, flavanones, flavonoids. The spectra of the samples show that all bee products, except propolis, have small peak or shoulder at 240–290 nm and 300–390 nm, what reveals similar chemical composition of bee pollen, beebread, royal jelly and honey. According to the literature, all flavonoids could be seen in this range of wavelength [47]. The spectra of propolis distinguished from other bee products. The absorption in propolis sample around 280 nm and at 320–330 nm indicates that propolis sample may have compounds belonging to flavanol class. Also, the band around 220–230 nm in propolis sample is attributed mainly to the aliphatic dienes. Furthermore, another common bands in the most samples are in the range of 200–240 nm and indicates presence of carboxylic acids. 

The UV-Vis spectrophotometry is assumed as complex analysis method and is limited by the specific difficulties in determining absorption peaks to certain system. For the proper identification and characterization of constituent of samples, UV-Vis results must be provided by other analytical techniques, for example liquid chromatography with mass spectrometry (LC-MS), gas chromatography with mass spectrometry (GC-MS) etc.

### 3.4. Mineral Content in the Tested Samples

The content of As, Ba, Ca, Cd, Co, Cr, Cu, Fe, K, Mg, Mn, Na, Ni, P, Pb, Se, Sr, V, and Zn was analysed in the bee pollen and other bee product samples by inductively coupled plasma mass spectrometer. The summary of the results is listed in Table 4. The minerals V, Ni, As and Se were not detected in the tested samples at all. Amount of the measured minerals varied depending on the sample type and botanical origin. Melissopalynological analysis of the samples was not performed, but different colour of tested pollen, beebread or honey undoubtedly refers to different botanical origin of these samples (Table 4). 

Bee pollen was distinguished by the highest amount of Ca (997–2455 mg/kg), while the amount of Ca was less than 612 mg/kg in the other samples. It also exhibited the presence of Sr, which was not detected in other samples at all. The first time Sr was reported by Kostic et al. in bee pollen samples from Serbia [48]. Bee pollen together with several samples of beebread also exposed the highest amount of P (2820–4840 mg/kg). Propolis differed from the rest set of samples by the highest amount of Fe (245.4–304.5 mg/kg), however, from the nutrition point of view, bee pollen and beebread would be better source of Fe (25.1–76.2 mg/kg) as pure propolis is not recommended for food consumption.

Bee pollen was also characterised by the high amount of Mg (644–1004 mg/kg) and the absence of Cr, which was found in other bee products. Interestingly to note, that Co was detected in 12 bee pollen samples out of 15, while other bee product samples did not reveal this element. Studies published in literature have shown that content of Co correlates with amount of vitamin B12 [49]. Evaluation of vitamin B12 was not under scope of this study, but there is a high chance that bee pollen may have higher content of vitamin B12 compared to other bee products, as cobalt is a key element in the structure of vitamin B12 (cyanocobalamin).

As beebread is made by fermenting bee pollen loads in the hive with a drop of honey and bee saliva, it was reasonable to expect similar mineral profile for both these products. However, as it was mentioned before, Sr was detected only in bee pollen samples, while Cu content was higher in the most of beebread samples (see Table 4). The presence of Cr in beebread and other products, except bee pollen, allows to assume that the origin of this element could be other than plants.

Unfortunately, some environment pollutants, such as Pb, Ba and Cd, were also detected in the tested samples. Pb was found in all samples and the amount was up to 0.433 mg/kg in bee pollen, beebread, honey and royal jelly, while propolis contained more than 20 times higher amount of Pb – up to 9.49 mg/kg. Ba was not detected in honey and beebread samples, except the BB1_LT, while Cd was not detected in all honey samples (see Table 4).

### 3.5. Clustering Analysis of the Data

**Figure 2:**
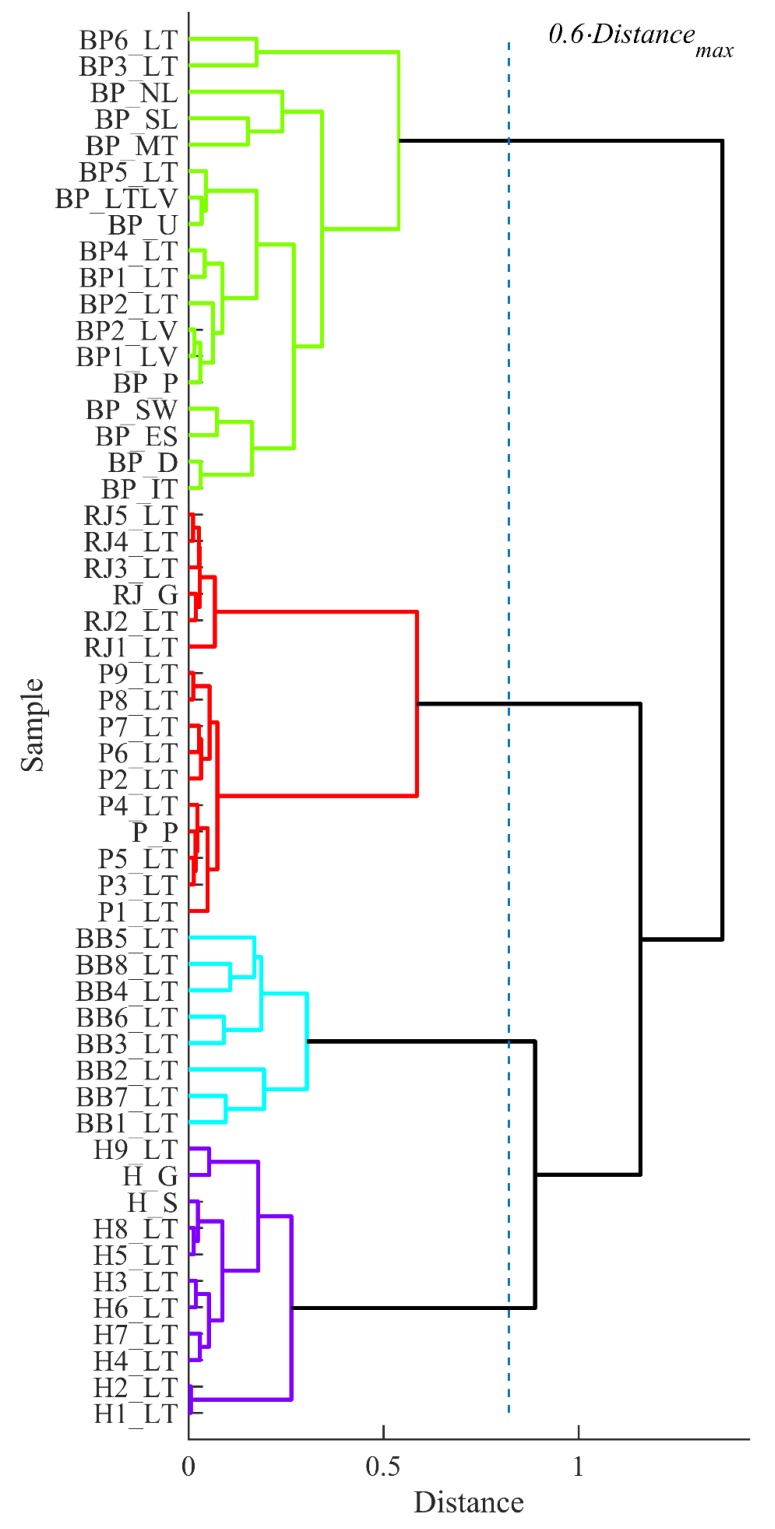
Clustering of the samples (HCA) by physicochemical properties (sample codes see in Table 1).

**Figure 3:**
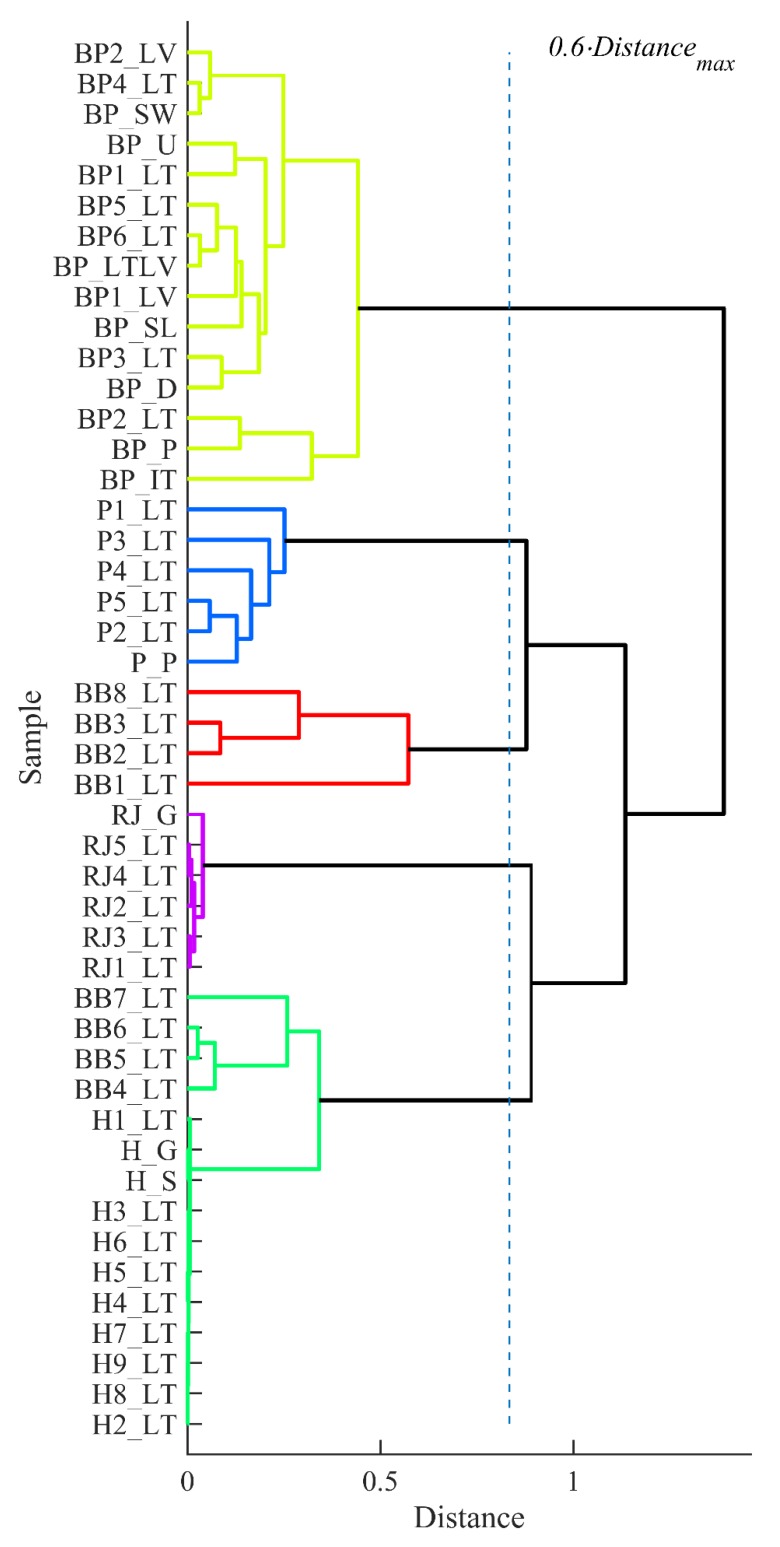
Clustering of the samples (HCA) by mineral content (sample codes see in Table 1).

Two dendrograms were built using HCA to group samples according to physicochemical properties and mineral analysis results (see Figure 2 and Figure 3). Calculated cophenetic correlation coefficients were 0.90 and 0.95 for constructed dendrogam trees based on described data sets, respectively.

The clustering result of the samples according to the physicochemical characteristics was unambiguous. The dendrogram in Figure 2 showed four groups of bee products (with tree cut-off at 0.6 of maximum distance), i.e., honey group, beebread group, propolis together with royal jelly group and bee pollen group, which exhibited the highest distance from others. The clustering of the samples according to mineral content formed five distinct groups cutting the dendrogram tree at 0.6 of maximum distance presented in Figure 3. Similar to previous data, bee pollen samples formed a separate group with the highest distance (highest dissimilarity). Four beebread samples (BB1_LT, BB2_LT, BB3_LT and BB8_LT) formed individual cluster, while the rest of the beebread samples were more similar to the honey samples and therefore were assigned to the same group. 

## 4. Conclusions

Physicochemical analysis of bee products contributed to a deeper characterisation of bee pollen and other bee products, namely, honey, beebread, propolis and royal jelly. According to the results, the highest values of pH, electrical conductivity and content of soluble solids were showed by bee pollen. The results of this research revealed that measured refractive index of tested bee products samples had no significant difference. The highest value of oxidation-reduction potential value was determined in propolis samples. Spectrophotometric evaluation of bee products exposed that the highest total phenolic compound content and radical scavenging activity was determined in propolis samples. Spectrophotometric assays in the ultraviolet-visible (UV-Vis) region enabled identification and characterization of chemical composition of different bee products, but obtained absorption spectra characteristic to phenolic acids and their derivatives (flavones, flavanols, flavanones, flavonoids, etc.).

Various valuable minerals can be found in bee products. Study showed that bee products, especially bee pollen, can be a source of Fe, Ca, Mg, K, Zn and Cu in the human diet. Bee pollen distinguished from the other bee products by the absence of Cr, the presence of Co (0.011–0.100 mg/kg) and Sr (0.73–5.37 mg/kg) and the highest content of Ca (997–2455 mg/kg) and Mg (644–1004 mg/kg).

Hierarchical clustering analysis applied for the grouping of the tested samples according to the physicochemical properties and mineral content revealed that bee pollen formed separate group with the highest distance (highest dissimilarity) from the other samples in both cases.

## Figures and Tables

**Table 1:** Characterisation of tested samples.

Name	Bee Product	Location	Country	Collection Period	GPS Coordinates
1	2	3	4	5	6
BP_IT	Bee pollen	Bibbiena region	Italy	2018	43°42′N 11°49′E
BP_D		Alsgarde region	Denmark	Aug 2018	56°04′N 12°32′E
BP_SW		Hagfors region	Sweden	Aug 2018	60°02′N 13°39′E
BP_SL		Trnava region	Slovakia	Jun 2018	48°22′N 17°35′E
BP_P		Bialystok	Poland	Jul 2018	53°08′N 23°08′E
BP_ES		Valencia region	Spain	May 2018	39°28’N 0°22’W
BP_MT		Northern region, Mellieha	Republic of Malta	Aug 2018	35°57′N 14°21′E
BP_NL		South Holland, Gouda	The Netherlands	Aug 2018	52°0′N 4°42′E
BP1_LT		Šiauliai region, Kuršėnai	Lithuania	Aug 2018	55°59’N 22°55’E
BP2_LT		Zarasai	Lithuania	2018	55°43’N 26°15’E
BP3_LT		Prienai	Lithuania	2018	54°37’N 23°56’E
BP4_LT		Kaunas	Lithuania	2018	54°53’N 23°53’E
BP5_LT		Radviliškis	Lithuania	Jul 2018	55°49’N 23°31’E
BP6_LT		Biržai and Panevėžys region mix	Lithuania	2018	55°44’N 24°22’E
BP1_LV		Saldus region	Latvia	Jun 201	56°40′N 22°12′E
BP2_LV		Alūksne region	Latvia	Jul 2018	57°23′N 27°6′E
BP_U		Volyn region	Ukraine	2018	50°44′N 25°21′E
BP_LTLV		Mix of Lithuanian and Latvian		2018	
BB1_LT	Beebread	Kaunas region	Lithuania	2018	54°55’N 23°57’E
BB2_LT		Kaišiadorys region	Lithuania	2018	54°52’N 24°26’E
BB3_LT		Šilutė region	Lithuania	2018	55°21’N 21°28’E
BB4_LT		Pasvalys region	Lithuania	2018	56°04’N 24°23’E
BB5_LT		Vilnius region	Lithuania	2018	54°43’N 25°22’E
BB6_LT		Skuodas region	Lithuania	2018	56°16’N 21°31’E
BB7_LT		Pakruojis region	Lithuania	2018	55°58’N 23°52’E
BB8_LT		Prienai region	Lithuania	2018	54°37’N 23°56’E
**1**	**2**	**3**	**4**	**5**	**6**
H2_LT	Honey	Vilkaviškis region, Švitrūnai	Lithuania	Jul 2018	54°38′N 22°52′E
H3_LT		Vilkaviškis region, Pilviškiai	Lithuania	Jun 2018	54°43′N 23°13′E
H4_LT		Panevėžys	Lithuania	Aug 2018	55°43’N 24°21’E
H5_LT		Prienai	Lithuania	Jul 2018	54°37’N 23°56’E
H_S		Sicilia (*Sulla coronaria* honey)	Italy	2018	37°49’N 15°16’E
H_G		Crete	Greece	2018	35°12′N 24°54′E
H6_LT		Anykščiai region	Lithuania	Jul 2018	55°31’N 25°06’E
H7_LT		Vilnius region	Lithuania	Jul 2018	54°41’N 25°16’E
H8_LT		Rokiškis	Lithuania	Jul 2018	55°58’N 25°34’E
H9_LT		Varėna	Lithuania	Jul 2018	54°13’N 24°34’E
P_P	Propolis	Bialystok	Poland	2018	53°7’N 23°10’E
P1_LT		Mažeikiai region	Lithuania	2018	56°19’N 22°19’E
P2_LT		Vilnius region	Lithuania	2018	54°49’N 25°19’E
P3_LT		Ignalina	Lithuania	2018	55°21’N 26°10’E
P4_LT		Marijampolė region	Lithuania	2018	54°45’N 23°15’E
P5_LT		Panevėžys and Šiauliai mix	Lithuania	2018	55°48’N 23°57’E
P6_LT		Šakių region, Pervazninkai	Lithuania	2018	55°02′N 22°43′E
P7_LT		Pasvalio region, Ustukiai	Lithuania	2018	56°04′N 24°21′E
P8_LT		Šalčininkų region, Didžiuliai	Lithuania	2018	54°15′N 25°37′E
P9_LT		Kretingos region, Baubliai	Lithuania	2018	55°49′N 21°24′E
BB1_LT	Beebread	Kaunas region	Lithuania	2018	54°55’N 23°57’E
BB2_LT		Kaišiadorys region	Lithuania	2018	54°52’N 24°26’E
BB3_LT		Šilutė region	Lithuania	2018	55°21’N 21°28’E
BB4_LT		Pasvalys region	Lithuania	2018	56°04’N 24°23’E
BB5_LT		Vilnius region	Lithuania	2018	54°43’N 25°22’E
BB6_LT		Skuodas region	Lithuania	2018	56°16’N 21°31’E
BB7_LT		Pakruojis region	Lithuania	2018	55°58’N 23°52’E
BB8_LT		Prienai region	Lithuania	2018	54°37’N 23°56’E
RJ_G	Royal jelly	Bradenburg region	Germany	2017	52°24’N 12°32’E
RJ1_LT		Kupiškis region, Lukonys	Lithuania	Jun 25, 2018	55°47’N 24°46’E
RJ2_LT		Kupiškis region, Lukonys	Lithuania	Aug 2, 2018	55°47’N 24°46’E
RJ3_LT		Pakruojis region, Oniūnai	Lithuania	Jul 16, 2018	55°50’N 24°49’E
RJ4_LT		Pakruojis region, Oniūnai	Lithuania	Aug 10, 2018	55°50’N 24°49’E
RJ5_LT		Pakruojis region, Oniūnai	Lithuania	Aug 13, 2018	55°50’N 24°49’E

**Table 2:** Physicochemical properties of bee pollen and other bee products (sample codes see in Table 1).

Sample	Sample Code	pH	Conductivity, µS/cm	ORP, mV	Brix, %	RI	NaCl, %
1	2	3	4	5	6	7	8
**Bee pollen**	BP_IT	4.32	744.6	100.20	31.84	1.343	6.34
	BP_D	4.40	757.0	85.42	27.36	1.343	6.26
	BP_SW	4.57	786.0	195.92	29.68	1.343	7.08
	BP_SL	4.53	836.4	94.17	25.84	1.344	6.66
	BP_P	5.05	577.4	103.84	25.52	1.344	8.84
	BP1_LT	4.90	573.6	187.30	24.08	1.343	9.24
	BP2_LT	4.91	546.0	107.72	24.64	1.343	10.36
	BP3_LT	4.96	476.6	155.08	22.56	1.344	10.94
	BP4_LT	5.22	456.6	157.88	30.80	1.343	10.14
	BP5_LT	5.09	699.4	124.99	28.16	1.343	10.46
	BP6_LT	4.63	444.2	69.10	24.64	1.344	10.68
	BP1_LV	5.02	689.0	100.05	28.56	1.344	10.76
	BP2_LV	4.98	667.4	106.81	26.16	1.345	10.00
	BP_U	5.00	645.2	123.58	29.44	1.344	11.24
	BP_LTLV	4.80	622.4	152.60	27.36	1.344	11.36
	BP_ES	4.34	731.0	162.83	28.24	1.343	5.66
	BP_MT	4.30	800.8	71.88	28.32	1.341	5.96
	BP_NL	4.43	455.0	122.44	25.44	1.343	6.94
	SD^a^	0.005	0.67	0.58	0.046	0.001	0.045
**Descriptive statistics**	Mean^b^ (LT)	4.95	533	114	28.0	1.343	10.30
	SD (LT)^c^	0.20	96	30	2.2	0.001	0.59
	Mean (all samples)	4.75	639	123	27.2	1.343	8.8
	SD (all samples)^c^	0.31	127	37	2.5	0.001	2.1
	Min^d^ (all samples)	4.30	444.2	69.10	22.56	1.341	5.66
	Max^e^ (all samples)	5.22	836.4	195.92	31.84	1.345	11.36
**Beebread**	BB1_LT	4.12	229.8	77.23	18.48	1.344	5.84
	BB2_LT	4.11	281.6	95.40	20.00	1.344	6.84
	BB3_LT	4.44	294.0	85.56	20.08	1.342	5.24
	BB4_LT	4.32	251.0	95.74	19.84	1.342	6.24
	BB5_LT	4.37	312.6	96.26	18.64	1.343	6.04
	BB6_LT	4.33	276.0	100.45	19.92	1.344	6.34
	BB7_LT	4.23	206.8	99.17	17.44	1.343	6.24
	BB8_LT	4.28	257.8	92.78	17.36	1.344	6.40
	SD	0.005	0.86	0.59	0.045	0.001	0.05
**Descriptive statistics**	Mean (all samples)	4.28	264	92.8	19.0	1.343	6.15
	SD (all samples)	0.12	35	7.8	1.2	0.001	0.47
	Min (all samples)	4.11	206.8	77.23	17.36	1.342	5.24
	Max (all samples)	4.44	312.6	100.45	20.08	1.344	6.84
**1**	**2**	**3**	**4**	**5**	**6**	**7**	**8**
**Honey**	H1_LT	4.30	146.6	77.67	16.88	1.347	1.94
	H2_LT	4.26	142.8	69.26	17.04	1.349	2.08
	H3_LT	3.81	155.8	106.98	16.96	1.349	2.14
	H4_LT	4.24	166.6	97.60	18.00	1.351	2.24
	H5_LT	4.17	158.8	107.91	17.12	1.350	2.36
	H_IT	4.30	181.6	100.70	17.44	1.351	2.28
	H_G	4.68	164.0	83.23	18.16	1.353	2.54
	H6_LT	3.72	162.6	99.55	17.52	1.350	2.24
	H7_LT	4.00	198.8	85.22	17.04	1.352	2.14
	H8_LT	4.12	179.4	87.09	18.24	1.351	2.42
	H9_LT	4.74	182.6	102.20	16.96	1.351	2.58
	SD	0.007	0.86	0.51	0.045	0.001	0.06
**Descriptive statistics**	Mean (LT)	4.15	166	93	17.31	1.350	2.23
	SD (LT)	0.30	18	14	0.50	0.001	0.20
	Mean (all samples)	4.21	167	93	17.40	1.350	2.26
	SD (all samples)	0.31	17	13	0.52	0.001	0.20
	Min (all samples)	3.72	142.8	69.26	16.88	1.348	1.94
	Max (all samples)	4.74	198.8	107.91	18.24	1.353	2.58
**Propolis**	P_P	4.19	327.8	162.10	13.84	1.337	5.84
	P1_LT	3.96	354.2	188.36	9.04	1.343	6.26
	P2_LT	4.06	256.4	104.14	8.96	1.336	6.34
	P3_LT	4.21	244.6	189.96	12.16	1.336	6.94
	P4_LT	3.95	388.6	161.74	10.88	1.338	6.60
	P5_LT	4.17	439.8	136.39	10.32	1.336	6.66
	P6_LT	4.02	401.0	107.77	10.64	1.337	6.28
	P7_LT	4.00	323.0	108.83	10.16	1.336	6.22
	P8_LT	3.98	366.8	93.93	12.40	1.337	5.94
	P9_LT	4.07	254.6	82.20	12.16	1.337	6.66
	SD	0.005	0.53	0.56	0.045	0.001	0.06
**Descriptive statistics**	Mean (LT)	4.04	337	130	10.8	1.337	6.43
	SD (LT)	0.09	71	41	1.3	0.001	0.30
	Mean (all samples)	4.06	336	134	11.1	1.337	6.37
	SD (all samples)	0.10	67	40	1.6	0.001	0.34
	Min (all samples)	3.95	244.6	82.20	8.96	1.336	5.84
	Max (all samples)	4.21	439.8	189.96	13.84	1.338	6.94
**Royal jelly**	RJ_G	4.04	263.4	98.16	13.36	1.339	3.48
	RJ1_LT	4.00	223.2	86.49	13.76	1.338	2.94
	RJ2_LT	3.57	230.2	83.48	12.24	1.337	3.46
	RJ3_LT	3.94	269.0	104.43	13.51	1.338	2.96
	RJ4_LT	3.95	222.8	94.27	14.56	1.339	3.44
	RJ5_LT	4.06	271.0	102.48	16.16	1.339	3.60
	SD	0.005	0.68	0.49	0.048	0.001	0.05
**Descriptive statistics**	Mean (LT)	3.90	243	94.2	14.1	1.338	3.28
	SD (LT)	0.19	25	9.3	1. 5	0.001	0.31
	Mean (all samples)	3.93	247	94.9	13.9	1.338	3.31
	SD (all samples)	0.18	24	8.5	1.3	0.001	0.29
	Min (all samples)	3.57	222.8	83.48	12.24	1.337	2.94
	Max (all samples)	4.06	271.0	104.43	16.16	1.339	3.60

^a^SD—combined standard deviation of measurements; ^b^Mean—average of measurand means; ^c^SD (*X* samples)—standard deviation of measurand means of the samples (where *X* are samples from Lithuania only or all samples); ^d^Min—the lowest value of measurand means; ^e^Max—the highest value of measurand means.

**Table 3:** Total phenolic compound content, total flavonoid content and radical scavenging activity of bee pollen and other bee products (sample codes, as seen in Table 1).

	Sample	Total Phenolic Compounds Content, mg RUE/10 g	Total Flavonoid Content, mg RUE/10 g	Radical Scavenging Activity, mg RUE/10 g
1	2	3	4	5
**Bee pollen**	BP_IT	48.28	24.12	25.08
	BP_D	49.27	25.17	21.77
	BP_SW	50.93	45.38	36.55
	BP_SL	43.56	34.51	21.73
	BP_P	49.00	40.06	27.48
	BP1_LT	50.68	42.93	30.69
	BP2_LT	50.85	29.26	27.76
	BP3_LT	44.70	10.68	16.27
	BP4_LT	55.04	46.85	27.20
	BP5_LT	33.14	21.29	23.06
	BP6_LT	41.66	10.82	16.29
	BP1_LV	51.12	42.51	33.43
	BP2_LV	53.84	48.31	39.40
	BP_U	39.03	22.21	17.69
	BP_LTLV	39.73	27.43	20.47
	BP_ES	53.29	30.68	39.40
	BP_MT	51.50	30.08	22.81
	BP_NL	44.43	38.39	34.29
	SD^a^	0.40	0.16	0.30
**Descriptive** **statistics**	Mean^b^ (LT)	46.0	27	23.5
	SD (LT)^c^	7.9	15	6.1
	Mean (all samples)	47.2	32	26.7
	SD (all samples)^c^	6.0	12	7.5
	Min^d^ (all samples)	33.14	10.68	16.27
	Max^e^ (all samples)	55.04	48.31	39.40
**Beebread**	BB1_LT	21.85	10.33	20.14
	BB2_LT	22.16	15.67	22.66
	BB3_LT	21.01	10.91	27.40
	BB4_LT	19.94	12.49	21.78
	BB5_LT	21.75	7.88	21.88
	BB6_LT	19.63	9.10	27.22
	BB7_LT	20.59	10.49	17.23
	BB8_LT	20.65	12.53	24.39
	SD	0.21	0.10	0.41
**Descriptive statistics**	Mean (LT)	20.95	11.2	22.8
	SD (LT)	0.92	2.4	3.5
	Min (all samples)	19.63	7.88	17.23
	Max (all samples)	22.16	15.67	27.40
**Honey**	H1_LT	4.07	2.26	3.86
	H2_LT	3.46	1.06	4.47
	H3_LT	3.68	1.98	2.22
	H4_LT	9.15	2.80	5.51
	H5_LT	7.03	5.22	3.77
	H_IT	3.43	0.91	2.73
	H_G	3.61	0.62	4.42
	H6_LT	10.18	3.99	8.26
	H7_LT	2.95	0.28	2.58
	H8_LT	7.58	2.29	3.03
	H9_LT	10.12	8.36	3.85
	SD	0.37	0.15	0.09
**1**	**2**	**3**	**4**	**5**
**Descriptive** **statistics**	Mean (LT)	6.1	3.1	4.20
	SD (LT)	3.1	2.4	1.74
	Mean (all samples)	5.6	3.6	4.06
	SD (all samples)	3.0	3.2	1.69
	Min (all samples)	2.95	0.28	2.17
	Max (all samples)	10.18	10.27	8.46
**Propolis**	P_P	87.12	10.01	28.62
	P1_LT	77.74	3.24	39.64
	P2_LT	68.03	5.92	25.37
	P3_LT	87.01	5.79	29.80
	P4_LT	92.74	9.67	31.78
	P5_LT	92.84	7.98	31.29
	P6_LT	80.81	6.58	19.69
	P7_LT	91.51	10.68	23.18
	P8_LT	99.85	9.26	22.10
	P9_LT	94.38	14.39	18.79
	SD	0.27	0.13	0.22
**Descriptive** **statistics**	Mean (LT)	87.2	8.1	26.9
	SD (LT)	9.9	3.3	6.8
	Mean (all samples)	87.2	8.4	27.0
	SD (all samples)	9.4	3.2	6.4
	Min (all samples)	68.03	3.24	18.79
	Max (all samples)	99.85	14.39	39.64
**Royal jelly**	RJ_G	22.49	13.20	7.05
	RJ1_LT	20.11	12.61	8.06
	RJ2_LT	16.44	10.34	5.16
	RJ3_LT	22.27	17.19	6.41
	RJ4_LT	23.14	16.31	7.61
	RJ5_LT	20.06	15.81	6.05
	SD	0.27	0.17	0.06
**Descriptive** **statistics**	Mean (LT)	20.4	14.5	6.7
	SD (LT)	2.6	2.9	1.2
	Mean (all samples)	20.8	14.2	6.7
	SD (all samples)	2.5	2.6	1.1
	Min (all samples)	16.44	10.34	5.16
	Max (all samples)	23.14	17.19	8.06

^a^SD—combined standard deviation of measurements; ^b^Mean—average of measurand means; ^c^SD (*X* samples)—standard deviation of measurand means of the samples (where *X* are samples from Lithuania only or all samples); ^d^Min—the lowest value of measurand means; ^e^Max—the highest value of measurand means.

**Table 4:** Total amount of phosphorus (P), macroelements and microelements in bee pollen samples, beebread, honey, propolis and royal jelly (expressed in mg of corresponding element in 1 kg of bee product, mg/kg) (sample codes see in Table 1).

Sample	Sample Code	P	K	Ca	Mg	Fe	Na	Mn	Zn	Cu	Sr	Cr	Co	Cd	Ba	Pb	Total Sum
1	2	3	4	5	6	7	8	9	10	11	12	13	14	15	16	17	18
**Bee pollen**	**BP1_LT**	3543	3124	2455	721.1	56.8	37.9	26.4	21.5	1.59	5.37	Nd^a^	0.026	0.072	0.775	0.269	9993
	**BP2_LT**	4375	3305	1203	633.5	46.1	46.1	30.7	27.8	Nd	0.73	Nd	0.100	0.349	0.650	0.237	9669
	**BP3_LT**	3986	2941	1844	642.8	53.2	44.7	23.0	25.1	5.90	1.44	Nd	0.016	0.034	0.798	0.309	9569
	**BP4_LT**	4039	2995	1719	715.7	52.3	41.0	26.5	22.8	1.88	0.89	Nd	0.011	0.105	1.05	0.381	9615
	**BP5_LT**	4456	3444	1760	927.0	66.9	34.3	18.5	23.7	Nd	2.09	Nd	0.039	0.007	1.06	0.248	10735
	**BP6_LT**	4266	3474	1830	995.4	50.0	41.0	26.4	20.3	Nd	2.21	Nd	Nd	0.014	0.951	0.253	10707
	**BP_LTLV**	4192	3494	1821	1004	56.4	38.9	26.4	20.8	0.55	2.05	Nd	0.017	0.039	0.928	0.148	10657
	**BP1_LV**	4032	3423	1885	644.1	63.1	31.3	27.0	23.5	0.44	1.71	Nd	0.040	0.082	0.956	0.277	10132
	**BP2_LV**	3218	3013	1576	915	57.0	37.9	31.6	24.7	0.48	0.92	Nd	0.018	0.141	0.896	0.197	8875
	**BP_IT**	2865	2766	1506	796.6	64.2	99.8	33.5	25.5	2.70	1.73	Nd	0.080	0.025	1.59	0.227	8164
	**BP_D**	3746	2947	1932	746.9	45.7	46.3	18.1	22.3	4.28	1.04	Nd	Nd	0.037	0.431	0.138	9510
	**BP_SW**	3460	2748	1768	837.7	53.5	37.7	29.6	23.3	1.41	0.99	Nd	Nd	0.112	0.903	0.249	8960
	**BP_SL**	2820	2400	997	644.1	76.2	39.0	18.2	28.7	2.31	1.67	Nd	0.022	0.065	0.946	0.211	7028
	**BP_P**	4841	3750	1487	881	53.6	24.5	66.3	31.7	nd	1.93	Nd	0.097	0.223	1.108	0.341	11139
	**BP_U**	3028	2682	1668	731.8	51.9	26.4	15.4	22.1	5.49	2.23	Nd	0.023	0.018	2.01	0.215	8235
	SD^b^	22	11	8.9	5.9	0.45	1.8	0.30	0.24	0.15	0.071		0.004	0.006	0.034	0.013	28
**Descriptive statistics**	Mean^c^ (LT)	3976	3131	1674	778	56.5	38.3	30	25.0	1.5	1.9		0.035	0.11	0.91	0.278	9713
	SD (LT)^d^	591	397	401	131	9.0	6.3	14	3.6	1.8	1.4		0.037	0.11	0.15	0.053	1175
	Mean (all samples)	3791	3100	1697	789	56.5	42	28	24.3	1.8	1.8		0.033	0.088	1.00	0.247	9532
	SD (all samples)^d^	602	365	326	127	8.0	17	12	3.1	2.0	1.1		0.033	0.091	0.37	0.065	1104
	Min^e^ (all samples)	2805	2395	996	625	45.04	23.84	15.34	20.04	0.00	0.64		0.000	0.006	0.40	0.117	7009
	Max^f^ (all samples)	4845	3759	2475	1009	76.40	100.26	66.49	31.99	6.05	5.63		0.105	0.370	2.02	0.391	11155
**1**	**2**	**3**	**4**	**5**	**6**	**7**	**8**	**9**	**10**	**11**	**12**	**13**	**14**	**15**	**16**	**17**	**18**
**Beebread**	**BB1_LT**	3349	1489	612	316.3	67.7	25.5	28.4	26.7	10.4	Nd	0.48	Nd	0.040	8.17	0.243	5933
	**BB2_LT**	2968	457	501	342.2	59.7	31.7	35.6	28.9	16.7	Nd	0.76	Nd	0.055	Nd	0.433	4442
	**BB3_LT**	2812	547	567	314	72.9	32.3	20.7	21.8	10.6	Nd	0.77	Nd	0.033	Nd	0.309	4399
	**BB4_LT**	1900	1154	597	316	41.4	27.7	7.2	17.4	7.3	Nd	0.35	Nd	0.016	Nd	0.235	4068
	**BB5_LT**	1796	946	548.7	409.1	48.2	24.2	10.8	13.5	6.6	Nd	0.47	Nd	0.018	Nd	0.147	3803
	**BB6_LT**	1615	933	566.1	343	53.9	24.6	11.0	13.9	5.6	Nd	0.43	Nd	0.018	Nd	0.150	3567
	**BB7_LT**	1363	2171	504.0	374.5	25.1	24.6	9.3	11.5	4.8	Nd	0.20	Nd	0.014	Nd	0.096	4488
	**BB8_LT**	2318	1434	572	342.1	40.5	33.0	29.1	42.7	8.2	Nd	0.33	Nd	0.033	Nd	0.192	4820
	SD	121	66	8.7	7.9	2.9	1.7	1.4	1.1	1.1		0.041		0.003	1.1	0.020	140
**Descriptive statistics**	Mean (all samples)	2265	1142	558	345	51	28.0	19	22	8.8		0.5		0.028	1.0	0.23	4440
	SD (all samples)	692	536	39	33	15	4.0	11	10	3.8		0.2		0.014	2.8	0.10	707
	min (all samples)	1270	430	493	300	22.92	22.68	6.72	10.78	4.40		0.187		0.012	0.00	0.081	3490
	max (all samples)	3432	2288	619	416	77.64	34.34	39.38	43.70	19.42		0.849		0.061	9.18	0.468	6091
**Royal jelly**	**RJ1_LT**	1760	2438	215	446	9.33	203	Nd	18.3	8.92	Nd	0.22	Nd	0.002	Nd	0.278	5098
	**RJ2_LT**	1743	2434	269	502	6.80	270	Nd	19.7	9.25	Nd	0.21	Nd	0.003	Nd	0.452	5255
	**RJ3_LT**	1805	2212	132.5	434	8.64	212.9	Nd	19.7	7.78	Nd	0.22	Nd	0.001	Nd	0.205	4833
	**RJ4_LT**	1558	2188	141.0	387	8.93	207.6	Nd	18.1	7.51	Nd	0.23	Nd	0.002	Nd	0.368	4517
	**RJ5_LT**	1759	2322	220	436	7.74	246	Nd	18.6	9.81	Nd	0.27	Nd	0.002	Nd	0.290	5020
	**RJ_G**	2246	3130	215.2	581	12.41	268.0	Nd	24.1	11.10	Nd	0.28	Nd	0.002	Nd	0.418	6488
	SD	45	22	8.6	14	0.49	8.1		0.56	0.38		0.015		0.000		0.036	84
**Descriptive statistics**	Mean (LT)	1725	2319	195	441	8.29	228		18.89	8.65		0.228		0.002		0.318	4945
	SD (LT)	101	112	54	41	1.05	28		0.91	0.95		0.025		0.001		0.096	277
	Mean (all samples)	1812	2454	199	464	9.0	235		19.8	9.06		0.237		0.002		0.335	5202
	SD (all samples)	220	327	50	65	1.9	30		2.1	1.30		0.032		0.001		0.095	643
	min (all samples)	1538.2	2165.4	127.1	381.6	6.31	194.2		17.33	7.24		0.209		0.001		0.189	4507
	max (all samples)	2266.0	3147.2	279.7	596.9	13.07	277.6		24.54	11.78		0.308		0.004		0.480	6530
**1**	**2**	**3**	**4**	**5**	**6**	**7**	**8**	**9**	**10**	**11**	**12**	**13**	**14**	**15**	**16**	**17**	**18**
**Honey**	**H1_LT**	109.3	556	52.2	27.2	1.24	13.2	Nd	1.63	1.84	Nd	0.18	Nd	Nd	Nd	0.257	763
	**H2_LT**	31.2	87.0	23.2	9.0	0.58	14.1	Nd	1.71	1.15	Nd	0.22	Nd	Nd	Nd	0.235	168
	**H3_LT**	79.2	120.7	31.2	15.4	1.58	12.8	1.08	1.63	2.03	Nd	0.54	Nd	Nd	Nd	0.138	2665
	**H4_LT**	57.3	271.3	25.6	11.4	1.24	10.9	0.77	2.89	1.70	Nd	0.24	Nd	Nd	Nd	0.217	383
	**H5_LT**	47.4	338.7	27.6	12.6	1.29	8.29	Nd	1.85	1.16	Nd	0.36	Nd	Nd	Nd	0.233	439
	**H6_LT**	126.1	82.2	19.6	13.2	1.09	11.7	Nd	5.15	1.72	Nd	0.47	Nd	Nd	Nd	0.218	261
	**H7_LT**	81.0	34.6	28.3	12.8	1.63	11.9	0.17	1.87	1.08	Nd	0.63	Nd	Nd	Nd	0.075	174
	**H8_LT**	102.9	125.8	25.3	12.7	1.36	11.4	Nd	1.08	1.10	Nd	0.29	Nd	Nd	Nd	0.192	282
	**H9_LT**	98.9	66.9	34.5	11.8	0.77	10.5	Nd	2.43	1.03	Nd	0.28	Nd	Nd	Nd	0.234	227
	**H_S**	39.7	168.8	26.1	8.17	0.43	19.6	Nd	2.03	1.98	Nd	0.11	Nd	Nd	Nd	0.171	267
	**H_G**	22.9	160.3	19.1	7.79	1.02	29.8	Nd	2.18	2.51	Nd	0.15	Nd	Nd	Nd	0.219	246
	SD	2.9	4.3	1.5	0.53	0.09	0.75	0.06	0.15	0.13		0.017				0.023	7.0
**Descriptive statistics**	Mean (LT)	82	187	29.7	14.0	1.20	11.6	0.23	2.3	1.42		0.36				0.200	329
	SD (LT)	30	164	9.2	5.0	0.34	1.8	0.40	1.2	0.39		0.15				0.059	178
	Mean (all samples)	72	183	28.4	12.9	1.11	14.0	0.18	2.22	1.57		0.32				0.199	316
	SD (all samples)	34	148	8.9	5.1	0.38	5.8	0.37	1.05	0.50		0.16				0.056	163
	min (all samples)	21.4	34.6	17.9	7.2	0.379	7.28	0.000	1.04	0.99		0.090				0.069	165
	max (all samples)	130.6	565.2	54.0	28.2	1.720	30.63	1.161	5.29	2.93		0.655				0.292	780
**Propolis**	**P1_LT**	512	495	542	96.4	301.4	155.1	25.1	40.1	11.50	Nd	4.35	Nd	0.026	3.02	3.49	2190
	**P2_LT**	369	429	248	69.1	274.3	41.9	24.2	38.7	6.85	Nd	12.13	Nd	0.037	8.13	7.24	1528
	**P3_LT**	375	252	235	31.0	252.5	36.1	16.5	102.1	2.36	Nd	5.39	Nd	0.012	9.59	9.49	1326
	**P4_LT**	358	545	476	98.4	304.5	124.5	15.0	31.9	3.01	Nd	4.73	Nd	0.040	9.29	5.84	1976
	**P5_LT**	393	360	405	83.4	234.2	26.9	23.3	40.8	14.31	Nd	11.13	Nd	0.041	10.20	5.31	1607
	**P_P**	244	242	254	69.3	245	28.2	28.8	52.4	8.53	Nd	4.32	Nd	0.072	8.61	4.60	1190
	SD	11	11	15	5.0	7.6	2.4	1.6	2.7	0.48		0.36		0.003	0.55	0.19	34
**Descriptive statistics**	Mean (LT)	401	416	381	76	273	76	20.8	50.7	7.6		7.5		0.032	8.1	6.3	1726
	SD (LT)	60	108	127	26	29	54	4.6	26.9	4.9		3.5		0.013	2.8	2.1	326
	Mean (all samples)	375	387	360	75	269	69	22.2	51	7.8		7.0		0.039	8.1	6.0	1636
	SD (all samples)	81	118	125	24	29	53	5.2	24	4.4		3.4		0.019	2.5	2.0	360
	min (all samples)	233.7	235.2	216.5	27.2	226.4	24.61	14.39	30.06	2.18		3.88		0.010	2.87	3.21	1168
	max (all samples)	517.7	557.7	552.1	104.0	309.2	158.10	31.26	107.50	14.72		12.19		0.075	10.77	9.69	2203

Note: Several bee pollen and propolis samples, which are listed in Table 1, were not tested for mineral content due to the lack of samples. ^a^Nd—not detected; ^b^SD —combined standard deviation of measurements; ^c^Mean - average of measurand means; ^d^SD (*X* samples)—standard deviation of measurand means of the samples (where X are samples from Lithuania only or all samples); ^e^Min—the lowest value of measurand means; ^f^Max—the highest value of measurand means.
